# Effect of Methionine Analogues on Growth Performance, Serum Biochemical Parameters, Serum Free Amino Acids and Rumen Fermentation of Yaks

**DOI:** 10.3390/ani12223175

**Published:** 2022-11-16

**Authors:** Xirui Zhang, Zizhen Zuo, Yao Liu, Chenxi Wang, Zhongli Peng, Jincheng Zhong, Ming Zhang, Haibo Wang

**Affiliations:** 1 College of Animal and Veterinary Sciences, Southwest Minzu University, Chengdu 610041, China; 2 Key Laboratory of Qinghai-Tibetan Plateau Animal Genetic Resource Reservation and Utilization, Sichuan Province and Ministry of Education, Southwest Minzu University, Chengdu 610041, China

**Keywords:** yak, methionine analogues, serum metabolite, free amino acid, ruminal fermentation

## Abstract

**Simple Summary:**

Yaks (*Bos grunniens*) are one of the most remarkable domestic animals; however, they generally suffer from malnutrition for almost 8 months of the year, which results in yaks displaying a circannual rhythm with seasonal changes. Yaks are ruminant animals whose utilization of dietary nitrogen efficiency is only about 15.9–19.0%; a large amount of N is excreted in feces and urine. A nutritional method could be taken to improve the utilization rate of dietary nutrients and regulate the synthesis of protein. Methionine makes up the first two limiting amino acids in a corn–soybean-meal-based diet for ruminants; therefore, we added the methionine analogues to the diet to investigate its role in yaks, eventually to find an efficient way to feed yaks.

**Abstract:**

This experiment was conducted to investigate the effects of methionine analogues 2-hydroxy-4-methylthio butanoic acid isopropyl ester (HBMi) on growth performance, nutrient apparent digestibility, serum metabolite, serum free amino acids, and rumen fermentation parameters of yaks. Twenty-four male Maiwa yaks (252.79 ± 15.95 kg) were randomly allocated to four dietary treatments: basic diet (CON), or three HBMi (MetaSmart (MS); Adisseo Inc., Antony, France) supplementation treatments: MS1 (5 g), MS2 (10 g), and MS3 (15 g). The results showed that the increase in the supplemented MS levels linearly increased the average daily gain (*p* < 0.05), while the serum alkaline phosphatase activity and malondialdehyde content were increased when yaks were fed with 15 g/d MS (*p* < 0.05). The diet supplemented with MS linearly increased the percentages of glutamic acid and proline, and linearly or quadratically decreased the percentages of isoleucine, phenylalanine, and valine (*p* < 0.05). Furthermore, supplementation of 10 g/d and 15 g/d MS increased ruminal microbial crude protein (*p* < 0.05). The ratio of acetate to propionate in the MS2 group was lower than those in CON and MS1 groups (*p* < 0.05). In summary, a diet supplemented with 10 g/d MS could be an effective way to improve the growth performance of fattening yaks without negative effects.

## 1. Introduction

The yak (*Bos grunniens*) is one of the most remarkable domestic animals in the world, which can adapt to the harsh environment at altitudes ranging from 3000 m to 5000 m because of their unique physiological characteristics [1]. There are approximately 16 million yaks worldwide, with 95% on the Qinghai–Tibetan Plateau in China [2]. Yak meat and milk have better quality, higher protein content, and higher polyunsaturated fatty acid concentrations than cattle from the lowland, for example, Holstein cattle [3,4]. Given the unique graze pattern, yaks generally suffer from malnutrition for almost 8 months of the year, which results in yaks displaying a circannual rhythm with seasonal changes [5]. The saying ‘strong in summer, weighty in autumn, thin in winter, and dead in spring’ is an accurate description of the growing and survival pattern of yaks across the Qinghai–Tibetan plateau [1]. 

Rumen is a unique protein metabolism site in ruminants. Ruminants obtain nitrogen (N) sources that are available for metabolism from dietary intake, microbial proteins, and endogenous N [6]. Rumen-degradable protein (RDP) and rumen-undegradable protein (RUP) are the two main classifications of dietary protein. RDP is degraded by rumen microbes, then synthesized into microbial protein or partly bypassed, whereas RUP directly bypasses the rumen. The digesta, escaping the stomach and reaching the small intestine, is disassembled and absorbed into the blood stream, and undigested proteins are excreted as feces. Microbial crude protein (MCP) and RUP, a part of endogenous crude protein synthesized in rumen, contribute to the passage of CP into the small intestine [7]. Diet is an important factor that influences the rumen microbiome [8]. 

Methionine (Met) and lysine (Lys) are the first two limiting amino acids in a corn–soybean-meal-based diet for ruminants [9]. Studies illustrated that dietary methionine supplementation can improve the performance of animals [10,11]. As Met could be directly metabolized in the rumen by the microbiome, various strategies such as rumen-protected Met and Met analogues have been developed to allow its direct absorption by the ruminant. The Met analogue 2-hydroxy-4-methylthio-butanoic acid isopropyl ester (HMBi) has played a role in ruminant nutrition [12]. Thus, we hypothesized that adding HBMi in yak basal diets will increase the growth performance and nutrient apparent digestibility; in addition, it provides balanced amino acids to yak small intestine absorption. MetaSmart (MS, Adisseo Inc., Antony, France), as a product of HMBi, is recommended at 1.9 to 2.5 g/kg dry-matter intake (DMI) in the ruminant [13,14]; hence, in the present trial, three gradients of MS (5 g/d, 10 g/d, and 15 g/d) were fed to yaks to evaluate growth performance, nutrient apparent digestibility, blood metabolites, and ruminal fermentation parameters.

## 2. Materials and Methods

### 2.1. Animals, Diets, and Experimental Design

All procedures in this study were approved by the Animal Care Committee of Southwest Minzu University (Protocol number: SMU 202106010). The experiment was conducted at Shengyuan yak husbandry Co., Ltd. in Xiaojin county, Aba Prefecture, Sichuan Province (102°59′ E, 30°35′ N, altitude at 2500 m). The temperature of the barn during the experiment was 5–12 °C. Twenty-four male Maiwa yaks (age: 4 years; live weight: 252.79 ± 15.95 kg) were randomly allocated to 1 of 4 dietary treatments according to BW. The BWs were sequenced from light to heavy, and then the randomization sequence was produced using Excel, with each group containing 6 replicates as follows: basic diet (CON) or three HBMi supplementation treatments—MS1 (5 g), MS2 (10 g), and MS3 (15 g). The MS was added into the total mixed ration, and supplied as a dry powder consisting of 57% HMBi, which in turn was 78% Met equivalent, of which 50% was absorbed through the rumen wall [15]; therefore, for each 10 g of MS, the yak received 2.22 g of Met. The proportions and chemical compositions of the basic diet are listed in Table 1. The trial lasted for seventy days followed by a ten-day adaptation. All animals were treated for internal and external parasites and vaccinated for common infectious diseases before the experiment started. Every yak was tethered separately and fed twice a day (7:30 and 15:30), the total mixed ration was offered ad libitum, and water was given uniformly after feeding. 

### 2.2. Sample Collection

Live body weights were recorded prior to the morning feeding at the 1st day and 70th day to calculate average daily gain (ADG). Feed and orts were recorded daily to calculate DMI. The ratio of DMI to ADG was used to define the feed conversion ratio (FCR). At the 70th day, before morning feeding, blood samples were collected from the jugular vein and centrifuged at 3000× *g* for 10 min at 4 °C to separate serum. 

Feed and orts were collected from the day 67 to day 69. At the same time, feces were collected using the total feces collection method, 20 mL 10% tartaric acid was added to 200 g fresh feces from each yak to prevent ammonia N volatilization, and they were stored at −20 °C for further analysis.

At 3 h after feeding, the rumen liquid was collected using an oral tube. The first 50 mL of the rumen liquid was discarded to avoid saliva effects. Additionally, about 60 mL of the rumen liquid was then extracted and stored in cryogenic vials after being filtered using 4 layers of sterilized gauze. The samples were placed into liquid nitrogen, and then brought back to the laboratory and stored in a −80 °C refrigerator for subsequent analysis.

### 2.3. Chemical Analysis 

Feed and feces samples were ground to pass through a 1 mm sieve. The dry matter (DM), CP, and ash of feed and feces were determined according to the procedure outlined in AOAC [16]. Acid detergent fiber (ADF) and neutral detergent fiber (NDF) were determined using the Van Soest’s method by a filter bag [17]. 

### 2.4. Serum Biochemical Parameters and Antioxidant Activity Analysis

Serum biochemical parameters and antioxidant activity analysis were analyzed following the procedure of our previous study [18]. Briefly, after the samples thawed at 4 °C, the contents of alanine aminotransferase (ALT), aspartate aminotransferase (AST), total protein (TP), albumin (ALB), creatinine (CERA), carbamide (UERA), uric acid (UA), glucose (GLU), total cholesterol (T-CHO), triglyceride (TG), and alkaline phosphatase (ALP) were determined using Hitachi 3100 (Hitachi Co., Tokyo, Japan). Globulin (GLB) content was calculated based on TP and ALB contents. Total superoxide dismutase (T-SOD) activity, total antioxidant capacity (T-AOC), glutathione peroxidase (GSH-Px), and malondialdehyde (MDA) content in serum were analyzed using a commercially available kit (Nanjing Jiancheng Bioengineering Institute, Nanjing, China) following the manufacturer’s instructions. The levels of insulin (INS) and insulin-like growth factor-1 (IGF-1) in serum were analyzed using commercially available kits (Jiangsu Jingmei Biotechnology Institute, Yancheng, China) following the manufacturer’s instructions.

### 2.5. Serum Free Amino Acids

The concentrations of Met, Lys, valine (Val), leucine (Leu), isoleucine (Ile), phenylalanine (Phe), tryptophan (Trp), histidine (His), arginine (Arg), glycine (Gly), alanine (Ala), serine (Ser), proline (Pro), asparagine (Asn), ornithine hydrochloride (Orn), aspartic acid (Asp), glutamine (Gln), glutamic (Glu), tyrosine (Tyr), and threonine (Thr) in the serum were determined using high-performance liquid chromatography (ACQUITY, Waters, Milford, MA, USA) with the column (ACQUITY, Waters, Milford, MA, USA). Metabolite extraction and a computer test were determined following the procedures of previous studies [19,20].

### 2.6. Rumen Fermentation Parameter

The NH_3_-N concentration was analyzed using the alkaline phenol hypochlorite method [21]. The MCP was analyzed using the bicinchoninic acid kit (Solarbio life sciences, Beijing, China) following the procedures described by the previous study [22]. Volatile fatty acids (VFA) were measured by a gas chromatograph (Agilent 6890N, Santa Clara, CA, USA) equipped with a column (DB-WAXETR, 30 m × 1.0 µm × 0.53 mm, Agilent, Santa Clara, CA, USA) following our previous method [23]. Briefly, the 2-ethylbutyric acid (2EB) was used as an internal standard, and the calculation was carried out by an internal standard correction quantitative method. The specific operation parameters are as follows: the carrier gas was N_2_, the split ratio was 40:1, the flow rate was 2.0 mL/min, the average linear velocity was 38 cm/s, and the column pressure was 11.3 psi. The programmed temperature was adopted, the initial temperature was 120 °C (3 min), and the temperature was increased to 180 °C (1 min) at 10 °C/min. The hydrogen flame detector temperature was 250 °C. The H_2_ flow rate was 40 mL/min, air flow rate was 45 mL/min, column flow rate was 45 mL/min, the inlet temperature was 210 °C, and the injection volume was 0.6 μL.

### 2.7. Statistical Analysis

Data were analyzed by the one-way ANOVA program in SAS (SAS Inst. Inc., Cary, NC, USA, 2002); the Duncan multiple range test was used for the statistical analysis of all differences among group means. Orthogonal polynomials were used to analyze the linear and quadratic trend in the indexes with the addition of MS. The results were expressed by average, and the variation degree of each treatment was expressed by the standard error of mean (SEM). Differences for comparisons were considered to be significant when *p* < 0.05 and tendencies included 0.05 ≤ *p* < 0.10.

## 3. Results

### 3.1. Growth Performances

The growth performance of the yaks is presented in Table 2. The ADG of yaks was linearly increased with the increase in MS supplemental level (*p* < 0.05); compared to yaks in the CON group, the ADG of yaks in the MS1, MS2, and MS3 groups increased by 6.58%, 13.16%, and 18.42%, respectively. The FCR demonstrated quadratic decreasing trends (*p* = 0.075). No significant differences were found among treatments for the IBW, FBW, and DMI (*p* > 0.05). 

### 3.2. Nutrient Apparent Digestibility

As shown in Table 3, no significant differences were found among treatments for nutrient (OM, CP, NDF, ADF, and ADE) apparent digestibility.

### 3.3. Serum Biochemical Indexes

The serum biochemical indexes are shown in Table 4. Serum ALP activity was linearly increased (*p* < 0.05) with the increase in MS supplemental level, and the activity of ALP in MS3 was significantly higher than other groups (*p* < 0.05). Diet supplementation with MS had no significant differences in ALT and AST activities, and TG, GLB, BUN, TP, ALB, GLB, CREA, T-CHO, UA, and UREA contents (*p* > 0.05).

### 3.4. Serum Antioxidant Indexes and Growth Hormone

As shown in Table 5, different MS supplemental levels in the diet had no significant differences in serum T-SOD, T-AOC, and GSH-Px contents. The serum MDA content was linearly and quadratically increased with the increase in MS supplemental level (*p* < 0.05), and the MDA contents in MS3 were significantly higher than the other groups (*p* < 0.05). No significant differences were found among treatments for serum INS and IGF-1 levels (*p* > 0.05).

### 3.5. Serum Free Amino Acids

The contents of serum free amino acids are presented in Table 6. For serum nonessential amino acids (AA), the percentages of Glu and Pro increased linearly with the increase in MS supplemental level (*p* < 0.05), and the percentages of Glu in the MS2 and MS3 groups were higher than that in the CON group; the percentage of Pro in the MS3 group was higher than that in the CON group. For serum essential AA, the percentage of Arg decreased quadratically with the increase in MS supplemental level (*p* < 0.05), and the percentages of Arg in the MS1 and MS2 groups were lower than that in the CON group. The percentages of Ile and Phe decreased linearly with the increase in MS supplemental level (*p* < 0.05), and the percentages of Phe in the MS2 and MS3 groups were lower than that in the CON group. The percentage of Val decreased linearly and quadratically with the increase in MS supplemental level (*p* < 0.05). The percentage of Met tended to increase linearly with increasing MS supplemental level (*p* = 0.051).

### 3.6. Ruminal Fermentation Parameters

The ruminal fermentation parameters of yaks are shown in Table 7. The MCP in the MS2 and MS3 groups were significantly higher than that in the CON group (*p* < 0.05). The percentage of acetate and the ratio of acetate to propionate decreased linearly with the increase in MS supplemental level (*p* < 0.05), and the ratio of acetate to propionate in the MS2 group was significantly lower than that in the CON group (*p* < 0.05).

## 4. Discussion

Growing evidence suggests that Met is not only a building block of protein synthesis [24], but also influences the optimum growth, reproduction, lactation and maintenance of ruminants [25]. However, it is not possible to synthesize Met in ruminant hosts, so it has to be provided by feed-degradable protein and ruminal microbes [26]. Many studies have reported that the addition of Met may improve growth and nutritional digestibility, while Met deficiency frequently restricts the growth of ruminants [27]. In this study, the ADG of yaks in experimental groups increased linearly with the increase in MS supplemental level. Similar to our study, research showed that diet supplementation with RP-Met and RP-Lys improved the ADG of Holstein steers [28]. The IGF-1, primarily produced in liver, plays a vital role in growth regulation, development, and metabolism in cattle [29]. In this study, the similar IGF-1 concentration among the treatments indicated that MS supplementation did not affect the growth hormones of yaks. In this study, diet supplemented with MS had no effects on the nutrient digestibility of yaks. Contrary to our results, a study found that the digestibilities of DM, OM, and CP in Rahmani lambs were improved by supplementing RP-Met in diets [26]; the reason may be the different species and physiological stage.

BUN was produced by protein hydrolysis and amino acid metabolism, and its content could reflect the animal’s protein metabolism in the body and the amino acid balance of diets [30]. BUN content, which is determined by the absorption of amino acids in the small intestine, will increase if the limiting amino acids in the body are deficient or imbalanced [31]. In this study, no significant difference in serum TP, ALB, BUN, UA, and CREA was found among the treatments, indicating that all the amino acids in the diets were enough and balanced; the biochemical indexes in our study were in a similar range compared to a study where a diet containing protected methionine did not affect the metabolic and hematological indicators of beef cattle [32]. In ruminant animals, the increase in ALT activity has been proved to be associated with liver damage, while the increase in AST activity could indicate a growing intensity of metabolic changes [33]. In addition, the activity of ALP is related to metabolic syndrome and cardiovascular disease [34]. In this study, diet supplementation with MS did not affect the activities of serum ALT and AST, while the activity of serum ALP in the MS3 group was higher than that in other groups, indicating that excessive addition of MS may cause the metabolic burden of yaks. Contrary to our results, a study on Cashmere goats found that diet supplementation of RP-Met did not affect ALP in comparison to control groups at 30 days and 60 days [35]; the differences in results might be due to the different species and the environment.

The serum GLU concentration changes with the increase or decrease in dietary protein level in calves [36,37]. Similarly, diet supplementation with RP-Met could increase blood GLU concentration in dairy cows [38]. Consistent with the previous study, the serum GLU content of yaks in the MS supplementation groups tended to increase linearly than that in the CON groups. It was speculated that the supplementation of 10 g/d and 15 g/d MS in the diet may promote the metabolism and synthesis of glycogen in the liver of yaks and increase the serum GLU content.

Glutathione is a common antioxidant substance in the body which helps the body to remove harmful free radicals and alleviate the damage caused by various inflammatory reactions [39]. As a prerequisite substance for the synthesis of glutathione, glutamate plays an important role in improving the body’s antioxidant performance and regulating the surface [40]. Studies have shown that glutamine, a glutamate derivative, can improve the intestinal physiological function of young mammals [41] and enhance the body’s immunity and antioxidant capacity [42]. Although, in this study, the serum Glu content of the MS3 group was significantly higher than that of the control group and the Glu content showed a linear decreasing trend with the increase in the addition of MS. The addition of MS had no significant effect on SOD, GSH-Px, and T-AOC in the yak serum in the early stage of this study. These results indicated that the GSH metabolism pathway of yaks was not affected in this study, so the addition of MS did not improve the antioxidant performance of the yaks. It is speculated that the reasons may be due to the species and age. The physiological status of yaks aged about 4 years was too different from that of calves in this study, which resulted in different experimental results.

Beyond being a protein source of amino acids, Met is also beneficial to improving the body’s antioxidant capacity. Firstly, Met and cysteine (Cys) residues in proteins can scavenge reactive oxygen species, thereby protecting proteins and other macromolecules from oxidative damage [43]. Secondly, Met is the precursor of substances that play an important role in the body’s antioxidant protection system, such as taurine [44], and Cys derived from Met is a component of the intracellular antioxidant glutathione [45]. MDA is a soluble degraded product of lipids [46] and an indicator of lipid peroxidation [47] that indicates the oxidative damage of cells [48]. In this study, no significant effect was found on the levels of serum antioxidant indexes among the treatments, while the content of MDA increased with the MS3 supplementation. A study has shown that Met restriction could decrease mitochondrial oxygen radical generation and oxidative damage of rats [49], suggesting that excessive Met may cause lipid peroxidation and further produce MDA, which may further affect the health status of yaks [50]. 

The absorption and utilization of Met can be determined by measuring the content of free amino acids in the serum, which has been shown to be positively correlated with the level of amino acids in the small intestine [51,52]. In ruminant animals, when the amount of limiting amino acids absorbed in the small intestine increases, other essential amino acids in the serum may decrease as a result of increased protein synthesis [53,54]. In this study, both Val and Phe in the serum decreased with the supplementation of MS, indicating that diet supplementation with MS may improve the balance of amino acids in the serum and presumably increase the utilization of the above amino acids. Consistent with our results, researchers have reported that diet supplementation with 0.15% RP-Met or 0.15% RP-Met and 0.5% lysine hydrochloride increases the concentration of arterial serum Met of dairy cows [55], and diet supplementation with RP-Met decreases the concentration of plasma Val, Ile, and branched-chain amino acids in cows [56]. Free AA are interrelated in the metabolic pathways of nutrients; the increase in amino acid content in the diet will not only affect its concentration in plasma, but also affect the concentration of other amino acids in plasma [57]. The decrease in plasma amino acid content may be due to the increase in the absorbable Met in the small intestine, which improves the balance of intestinal amino acids and the ability of tissues to synthesize proteins using amino acids [58]. In this study, the content of serum free EAA, including Arg, Ile, Phe, and Val, in the MS2 and MS3 groups was lower than those in the CON and MS1 groups, suggesting that the addition of 10 g/d and 15 g/d MS to a yak’s diet can improve the ability of tissues to synthesize protein using amino acids. Consistent with our results, a study found that the addition of RP-Met to the diet of dairy cows increased the plasma concentration of Met, while the plasma concentrations of Gly, Ile, Leu, Thr, Tyr, Phe, and Val tended to decrease [51]. The results showed that Glu content increased linearly with the increase in MS supplemental level; little research has reported the relationship of Met and Glu. A study on zebrafish found that 3.0 mM Met exposure could increase both glutamate and ATP levels at synaptic clefts in the zebrafish brain by compromising both glutamatergic and purinergic signaling [59].

NH_3_-N is a digestive metabolite of protein and non-protein nitrogen in the rumen and an important nitrogen source for microbial protein synthesis [60]. The concentration of NH_3_-N in rumen fluid can indirectly reflect the balance between the digestion and utilization of dietary nitrogenous substances by rumen microorganisms and the synthesis of MCP by NH_3_-N [61]. In this study, rumen NH_3_-N concentration in each group ranged from 7.48 to 11.37 mg/dL; the concentration of NH_3_-N in rumen was above 5 mg/dl which can meet the growth needs of rumen microorganisms [62]. Rumen MCP, which is the main nitrogen source in ruminant life activities, can provide 60–85% of the total amino acids in the small intestine and then provide a large amount of protein for ruminant life activities [63]. In this study, MCP contents in the MS3 and MS2 groups were significantly higher than that in the CON group, which showed a linear increasing trend with the increase in MS supplemental level. The reason may be that the rumen-passing efficiency of MS added in the diet is 50%, and some of it will be released in the rumen and used by rumen microorganisms. In addition, as the first two limiting amino acids, Met release in the rumen can meet the nutritional needs of some rumen microorganisms, promote the growth and reproduction of rumen microorganisms, and improve the yield of MCP [9]. Rumen microorganisms produce a large amount of VFA after the fermentation of dietary carbohydrates, which can provide not only 70% to 80% of the animal’s energy, but also the amino acid carbon skeleton [64]. In this study, the percentage of acetate linearly decreased with the increase in MS level; our results are in contrast to the view that Met supplementation could increase the concentration of acetate in the rumen of dairy cows [65]. In fact, although the MS supplementation linearly decreased the percentage of acetate, the concentration of acetate in the MS supplementation groups (ranging from 42.21 to 45.09 mmol/L) was still higher than that in the CON group (41.06 mmol/L) numerically. The ratio of acetic acid to propionic acid in the rumen of yaks in the MS2 group was significantly lower than that in other treatment groups, indicating that the supplementation of 10 g/d MS could optimize the rumen fermentation level of yaks and the propionic-acid-dominated fermentation type was more conducive to the efficient production of yaks. 

## 5. Conclusions

Diet supplementation with MS linearly increased the growth performance of yaks, while it had no impact on the apparent nutrient digestibility. In addition, the supplementation of different levels of MS in the diet linearly increased the percentages of Glu and Pro, and decreased linearly or quadratically the percentages of Ile, Phe, and Val. Meanwhile, the serum ALP activity and MDA content were increased when yaks were fed with 15 g/d MS. Furthermore, the supplementation of 10 g/d and 15 g/d MS had a positive impact on the rumen fermentation parameters, indicated by the increased synthesis of MCP in the rumen. The findings of the current study indicate that diet supplementation with 10 g/d MS is an effective way for fattening yaks.

## Figures and Tables

**Table 1:** Ingredients and chemical composition of basic diets.

Item	
Ingredients (% DM ^1^)	
Corn meal	15.75
Soybean	4.50
Soybean meal	7.65
Rapeseed meal	2.25
Corn germ meal	3.60
Sprayed corn bran	4.50
Soybean hull	3.15
Molasses	2.25
Premix ^2^	1.35
Corn straw silage	55.0
Chemical composition (% DM)	
NE_g_ (MJ/kg)	3.52
NE_m_ (MJ/kg)	5.58
CP	13.13
NDF	43.92
ADF	15.63
OM	90.75

^1^ DM: dry matter; NE_g_: net energy for gain; NE_m_: net energy for maintenance; CP: crude protein; NDF: neutral detergent fiber; ADF: acid detergent fiber; OM: organic matter. ^2^ Every kilogram of mineral–vitamin premix contained: vitamin A, 2500 IU; vitamin D, 550 IU; vitamin E, 10 IU; Fe, 40 mg; Zn, 40 mg; Cu, 10 mg; Mn, 40 mg; I, 0.5 mg; Co, 0.2 mg; and Se, 0.2 mg.

**Table 2:** Effect of MetaSmart supplementation in diets on growth performance and economic benefit of yaks.

Items	Treatments ^1^				SEM	*p*-Value		
	CON	MS1	MS2	MS3		Treat	Linear	Quadratic
IBW (kg)	253.5	254.3	251.7	251.7	6.97	0.990	0.695	0.325
FBW (kg)	306.5	311.3	311.6	314.8	8.74	0.926	0.709	0.181
ADG (kg/d)	0.76	0.81	0.86	0.90	0.043	0.137	0.016	0.059
DMI (kg)	4.40	4.52	4.52	4.45	0.044	0.165	0.480	0.142
FCR	5.85	5.65	5.32	5.02	0.269	0.170	0.122	0.075

^1^ CON: yaks receiving a basic diet; MS1: yaks receiving a basic diet supplementation with 5 g MetaSmart (MS); MS2: yaks receiving a basic diet supplementation with 10 g MS; MS3: yaks receiving a basic diet supplementation with 15 g MS; SEM: standard error of mean; IBW: initial body weight; FBW: final body weight; ADG: average daily gain; DMI: dry-matter intake; FCR: feed conversion ratio = DMI/ADG.

**Table 3:** Effect of MetaSmart supplementation in diets on nutrient apparent digestibility of yaks.

Items	Treatments ^1^				SEM	*p*-Value		
	CON	MS1	MS2	MS3		Treat	Linear	Quadratic
OM (%)	50.41	54.15	55.41	53.65	3.569	0.803	0.326	0.892
CP (%)	52.98	52.05	53.91	53.20	4.125	0.991	0.619	0.638
NDF (%)	54.39	56.60	59.07	56.43	3.735	0.851	0.722	0.958
ADF (%)	31.54	34.76	34.83	31.39	5.559	0.948	0.931	0.962
ADE (MJ/d)	64.46	65.10	67.23	65.18	4.539	0.975	0.730	0.694

^1^ CON: yaks receiving a basic diet; MS1: yaks receiving a basic diet supplementation with 5 g MetaSmart (MS); MS2: yaks receiving a basic diet supplementation with 10 g MS; MS3: yaks receiving a basic diet supplementation with 15 g MS; SEM: standard error of mean; OM: organic matter; CP: crude protein; NDF: neutral detergent fiber; ADF: acid detergent fiber; ADE: apparent digestible energy.

**Table 4:** Effect of MetaSmart supplementation in diets on serum biochemical indexes of yaks.

Items	Treatments ^1^				SEM	*p*-Value ^2^		
	CON	MS1	MS2	MS3		Treat	Linear	Quadratic
ALT (U/L)	42.40	42.61	43.30	44.68	3.694	0.971	0.905	0.302
AST (U/L)	71.16	75.89	70.34	78.20	4.080	0.484	0.807	0.578
ALP (U/L)	97.60 ^a^	100.8 ^a^	111.0 ^a^	135.0 ^b^	7.441	0.016	0.039	0.468
TG (mmol/L)	0.40	0.42	0.47	0.40	0.033	0.379	0.505	0.519
GLB (g/L)	43.38	37.78	38.16	36.46	2.234	0.178	0.272	0.689
GLU (mmol/L)	4.11	4.44	4.77	4.60	0.181	0.095	0.084	0.398
BUN (mmol/L)	9.46	10.74	11.22	10.50	0.808	0.487	0.242	0.557
TP (g/L)	78.22	74.70	74.84	72.94	2.449	0.506	0.344	0.615
ALB (g/L)	34.84	36.92	36.68	36.48	0.769	0.251	0.427	0.026
CREA (umol/L)	152.3	160.4	167.6	156.4	8.160	0.603	0.362	0.284
T-CHO (mmol/L)	1.52	1.53	1.63	1.68	0.148	0.835	0.812	0.397
UA (umol/L)	23.56	23.94	28.22	29.84	3.149	0.425	0.686	0.661
UREA (mmol/L)	4.73	5.37	5.61	5.25	0.404	0.486	0.242	0.557

^1^ CON: yaks receiving a basic diet; MS1: yaks receiving a basic diet supplementation with 5 g MetaSmart (MS); MS2: yaks receiving a basic diet supplementation with 10 g MS; MS3: yaks receiving a basic diet supplementation with 15 g MS; SEM: standard error of mean; ALT: alanine aminotransferase; AST: aspartate aminotransferase; ALP: alkaline phosphatase; TG: triglycerides; GLB: Globulin; GLU: glucose; BUN: blood urea nitrogen; TP: total protein; ALB: albumin; CREA: creatinine; T-CHO: total cholesterol; UA: uric acid. ^2^ Means with different superscript letters in the same row are significantly different (*p* < 0.05).

**Table 5:** Effect of MetaSmart supplementation in diets on serum antioxidant indexes and growth hormone of yaks.

Items	Treatments ^1^				SEM	*p*-Value ^2^		
	CON	MS1	MS2	MS3		Treat	Linear	Quadratic
T-SOD (U/mL)	84.31	84.45	85.80	85.42	2.202	0.953	0.533	0.999
MDA (nmol/mL)	3.44 ^b^	2.56 ^b^	3.04 ^b^	4.64 ^a^	0.389	0.010	0.040	0.015
T-AOC (U/mL)	0.28	0.25	0.24	0.29	0.025	0.509	0.505	0.119
GSH-Px (U/mL)	19.77	21.71	29.49	31.57	5.835	0.438	0.284	0.524
INS (mIU/L)	31.71	28.36	27.14	28.88	1.157	0.066	0.744	0.159
IGF-1 (μg/mL)	37.08	34.23	34.38	36.59	2.029	0.669	0.888	0.476

^1^ CON: yaks receiving a basic diet; MS1: yaks receiving a basic diet supplementation with 5 g MetaSmart (MS); MS2: yaks receiving a basic diet supplementation with 10 g MS; MS3: yaks receiving a basic diet supplementation with 15 g MS; SEM: standard error of mean; T-SOD: total superoxide dismutase; MDA: malondialdehyde; T-AOC: total antioxidant capacity; GSH-Px: glutathione peroxidase; INS: insulin; IGF-1: insulin-like growth factor 1. ^2^ Means with different superscript letters in the same row are significantly different (*p* < 0.05).

**Table 6:** Effect of MetaSmart supplementation in diets on serum free amino acids of yaks (%).

Items	Treatments ^1^				SEM	*p*-Value ^2^		
	CON	MS1	MS2	MS3		Treat	Linear	Quadratic
NEAA								
Ala	8.78	8.82	10.84	9.95	0.718	0.171	0.073	0.635
Asn	4.16	4.20	3.73	4.27	0.240	0.401	0.476	0.729
Asp	0.66	0.66	0.77	0.78	0.063	0.369	0.185	0.962
Glu	4.37 ^a^	4.99 ^ab^	6.09 ^b^	6.15 ^b^	0.452	0.035	0.019	0.606
Gln	18.04	18.16	18.23	17.65	0.840	0.960	0.638	0.476
Gly	1.64	1.68	1.53	1.25	0.263	0.650	0.088	0.415
Orn	2.09	2.22	2.19	2.20	0.116	0.876	0.657	0.656
Pro	1.86 ^a^	1.89 ^a^	2.01 ^a^	2.69 ^b^	0.099	<0.001	0.017	0.086
Ser	2.45	2.89	2.37	2.75	0.177	0.169	0.842	0.407
Tyr	3.72	3.44	3.69	3.80	0.294	0.842	0.945	0.625
EAA								
Arg	8.99 ^b^	7.04 ^a^	7.53 ^a^	7.78 ^ab^	0.423	0.031	0.672	0.037
His	3.03	2.71	3.00	2.55	0.164	0.156	0.253	0.049
Ile	5.22	5.62	4.99	5.21	0.263	0.434	0.007	0.347
Leu	5.17	4.88	4.77	4.88	0.341	0.860	0.490	0.709
Lys	9.42	9.43	8.77	8.60	0.802	0.831	0.173	0.195
Met	1.97	2.29	2.74	3.01	0.263	0.078	0.051	0.522
Phe	4.17 ^b^	4.18 ^b^	3.48 ^a^	3.71 ^a^	0.150	0.012	0.034	0.578
Thr	2.87	3.30	2.74	3.09	0.299	0.578	0.174	0.223
Trp	2.94	2.87	2.83	2.94	0.233	0.984	0.326	0.422
Val	8.01	8.42	7.61	7.20	0.326	0.088	0.002	0.038

^1^ CON: yaks receiving a basic diet; MS1: yaks receiving a basic diet supplementation with 5 g MetaSmart (MS); MS2: yaks receiving a basic diet supplementation with 10 g MS; MS3: yaks receiving a basic diet supplementation with 15 g MS; SEM: standard error of mean; NEAA: nonessential amino acids; EAA: essential amino acids; Ala: alanine; Asn: asparagine; Asp: aspartic acid; Glu: glutamic acid; Gln: glutamine; Gly: glycine; Orn: ornithine hydrochloride; Pro: proline; Ser: serine; Tyr: tyrosine; Arg: arginine; His: histidine; Ile: isoleucine; Leu: leucine; Lys: lysine; Met: methionine; Phe: phenylalanine; Thr: threonine; Trp: tryptophan; Val: valine. ^2^ Means with different superscript letters in the same row are significantly different (*p* < 0.05).

**Table 7:** Effect of MetaSmart supplementation in diets on ruminal fermentation parameters of yaks.

Items	Treatments ^1^				SEM	*p*-Value ^2^		
	CON	MS1	MS2	MS3		Treat	Linear	Quadratic
NH_3_-N (mg/dL)	7.48	10.01	11.37	10.66	1.239	0.167	0.685	0.732
MCP (mg/mL)	0.54 ^a^	0.64 ^ab^	0.73 ^bc^	0.80 ^c^	0.052	0.019	0.097	0.376
T-VFA (mmol/L)	57.51	63.59	65.35	59.47	11.18	0.956	0.279	0.862
Acetate (%)	71.40	70.89	69.00	70.97	1.038	0.390	0.029	0.663
Propionate (%)	16.05	17.17	17.63	16.74	0.622	0.343	0.068	0.056
Butyrate (%)	8.46	7.19	8.77	7.80	0.987	0.697	0.060	0.546
Isobutyrate (%)	1.51	1.68	1.59	1.65	0.236	0.957	0.222	0.740
Valerate (%)	0.68	0.71	0.81	0.63	0.142	0.083	0.239	0.574
Isovalerate (%)	1.90	2.36	2.20	2.21	0.338	0.798	0.239	0.874
Acetate/Propionate	4.48 ^b^	4.46 ^b^	3.92 ^a^	4.25 ^ab^	0.125	0.017	0.022	0.084

^1^ CON: yaks receiving a basic diet; MS1: yaks receiving a basic diet supplementation with 5 g MetaSmart (MS); MS2: yaks receiving a basic diet supplementation with 10 g MS; MS3: yaks receiving a basic diet supplementation with 15 g MS; SEM: standard error of mean; MCP: microbial crude protein; T-VFA: total volatile fatty acid. ^2^ Means with different superscript letters in the same row are significantly different (*p* < 0.05).

## Data Availability

The data presented in this study are available in the article. Further information is available upon request from the corresponding author.
